# Recombination in West Nile Virus: minimal contribution to genomic diversity

**DOI:** 10.1186/1743-422X-6-165

**Published:** 2009-10-12

**Authors:** Brett E Pickett, Elliot J Lefkowitz

**Affiliations:** 1 Department of Microbiology, University of Alabama at Birmingham; Birmingham, AL 35294-2170, USA

## Abstract

Recombination is known to play a role in the ability of various viruses to acquire sequence diversity. We consequently examined all available West Nile virus (WNV) whole genome sequences both phylogenetically and with a variety of computational recombination detection algorithms. We found that the number of distinct lineages present on a phylogenetic tree reconstruction to be identical to the 6 previously reported. Statistically-significant evidence for recombination was only observed in one whole genome sequence. This recombination event was within the NS5 polymerase coding region. All three viruses contributing to the recombination event were originally isolated in Africa at various times, with the major parent (SPU116_89_B), minor parent (KN3829), and recombinant sequence (AnMg798) belonging to WNV taxonomic lineages 2, 1a, and 2 respectively. This one isolated recombinant genome was out of a total of 154 sequences analyzed. It therefore does not seem likely that recombination contributes in any significant manner to the overall sequence variation within the WNV genome.

## Background

The species *West Nile virus *(WNV) is a member of the family *Flaviviridae*, genus *Flavivirus*. West Nile virus is a positive-sense, single-stranded RNA virus that has 6 separate phylogenetically-distinct lineages which correlate well with the geographical point of isolation [1]. Sequence variation in positive-sense RNA viruses such as flaviviruses, can occur via single base changes and small insertions and deletions within the linear evolutionary pathway of the virus lineage [2-4]. In addition, larger scale sequence changes can occur via exchange of genetic information with other related viruses via the process of recombination [5,6]. Recombination has been detected in several members of the *Flaviviridae *family including: hepatitis C virus [7] and dengue virus [8,9]; and it has been hypothesized that West Nile virus would follow suit as more sequence data becomes available [10].

Homologous recombination in single-stranded RNA molecules occurs via a template-switch [11], also called copy-choice [12], mechanism. More specifically, when two positive-polarity, single-stranded RNA viruses belonging to the same species co-infect a single cell, a replicating viral RNA-dependent RNA polymerase (RdRp) can dissociate from the first genome and continue replication by binding to, and using a second distinct genome as the replication template. This dissociation process is thought to be initiated by the RdRp pausing or stalling at specific sequences or RNA structural elements [11,13,14]. The act of moving the RdRp complex from one "parental" genome to another yields a chimera "daughter" viral genome containing one fraction of the first "parental" genome and the other fraction of the second "parent" genome.

Such recombination events in natural sequences are difficult to detect in the wet-lab due to the sequence similarity that exists between parental and daughter sequences at any putative recombination breakpoint [15]. As a consequence of this fact, *in silico *techniques have been developed to assist in this endeavor. These algorithms function by comparing all possible combinations of three sequences at a time from a multiple sequence alignment to determine whether or not a nucleotide pattern signifying the presence of a recombination breakpoint exists within between any 3 sequences (two parental, and one recombinant).

To manually detect phylogenetic incongruencies between different regions of the aligned genomes, we analyzed portions of the MSA containing: the complete NS5 coding region, the NS5 coding region lacking the recombinant region, or only the region within the NS5 coding sequence that showed evidence of recombination. MrBayes was then used to reconstruct separate consensus phylogenetic trees using the parameters described below. The topologies of these three trees were compared to confirm recombination within the region.

## Results

### Phylogenetic Tree Reconstructions

**Figure 1:**
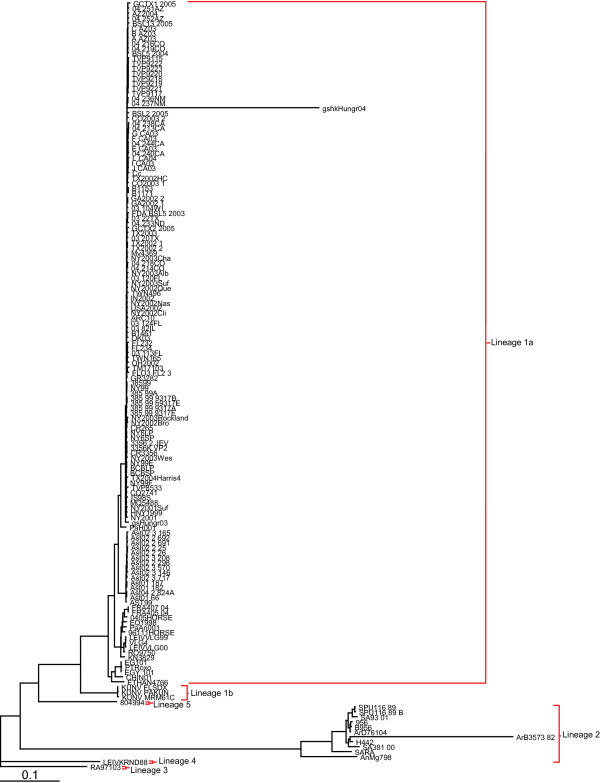
**Whole Genome Phylogenetic Tree**. Bayesian phylogenetic tree reconstruction of 154 whole genomic WNV (and Kunjin virus) sequences. The 6 distinct lineages are maintained and are delineated by red brackets. Branch lengths are proportional to distance (the number of nucleotide changes), and the distance scale for the number of changes is provided at the bottom of the figure.

When a Bayesian phylogenetic tree was reconstructed (figure 1), we found that the high number of sequences included in the present study maintained the 6-lineage topology present in trees published previously [1]. These lineages tend to correspond more with the general geographical location of isolates than with their temporal point of isolation or their host pathogenicity [16,17].

### Detection of Recombination in Whole Genome Sequences

In order to determine the extent of recombination within these whole genome WNV sequences, we used a suite of recombination detection programs including: RDP, GENECONV, BootScan, MaxChi, Chimaera, SiScan, PhylPro, LARD, and 3Seq; as well as the SplitsTree program. After comparing all 154 genomes (11,781 sequence comparisons), only one significant recombination event was detected (See additional file 1 and additional file 2 for the results from this analysis). For this single event, five of the nine algorithms detected significant recombination at the same location in the genome with p-values ranging from 4.936 × 10^-2 ^to 7.235 × 10^-8 ^(table 1). An additional algorithm detected recombination at the same location, although it lacked a statistically significant p-value. The location of the significant recombination breakpoint was in the NS5 coding region of the AnMg798 sequence isolated from a parrot in Madagascar in 1978. This sequence was marked as the daughter, or recombinant, with the major parent being the SPU116_89_B sequence isolated from a human in South Africa in 1989 and the minor parent being the KN3829 sequence isolated from a mosquito in Kenya in 1998. The lineages for these three sequences are 2, 2, and 1a respectively (table 2).

**Table 1:** Recombination Statistics

**Algorithm**	**Recombination P-value**	**NT Position**
RDP	4.936 × 10^-2^	9396-9630
GENECONV	2.033 × 10^-6^	9396-9630
BootScan	8.269 × 10^-5^	9396-9630
MaxChi	n/a	n/a
Chimaera	n/a	n/a
SiScan	3.600 × 10^-1^	9396-9630
PhylPro	n/a	n/a
LARD	7.235 × 10^-8^	9396-9630
3Seq	3.986 × 10^-5^	9396-9630

**Table 2:** Recombinant Sequences

**Name**	**Accession**	**Year**	**Location**	**Lineage**	**Host**	**Recombination**
SPU116_89_B	EF429197	1989	South Africa	2	Human	Major Parent
KN3829	AY262283	1998	Kenya	1a	Mosquito	Minor Parent
AnMg798	DQ176636	1978	Madagascar	2	Parrot	Daughter

### Confirmation of Recombination Event

**Figure 2:**
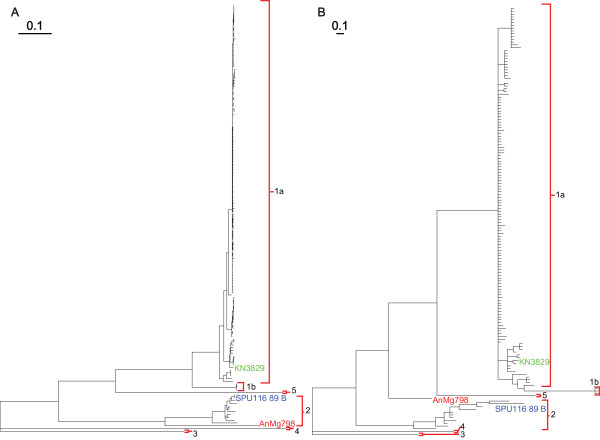
**Phylogenetic Trees Showing Recombination**. Shows the Bayesian consensus trees for (A) the NS5 coding region lacking the recombinant region and (B) only the recombinant region. The labels for all non-recombinant taxa were removed for clarity. The translocation of the AnMg798 sequence from the lineage 2 clade in panel A to the lineage 1a clade in panel B indicates the presence of recombinant sequence within this region. Major parent, minor parent, and daughter sequences are shaded in blue, green, and red respectively. Lineages are indicated as in figure 1. Branch lengths are proportional to distance (the number of nucleotide changes), and the distance scale for the number of changes is provided at the top each panel.

We confirmed the region identified as containing the recombination breakpoint by comparing the phylogenetic tree topologies of the entire NS5 coding region (data not shown) or the NS5 coding region without the recombinant region (figure 2A) (both of which produced topologies essentially the same as for the whole genome), to the putative recombinant region (figure 2B). For the recombinant region, we not only saw a change in the topology of the trees, but a decrease in the distance, or number of changes, which separates the daughter (AnMg798) and minor parent (KN3829) sequences from each other in the recombinant region. We realize that the recombinant region contains 235 nucleotide positions and that only 81 (34.45%) of those positions are parsimony-informative. Nevertheless, sufficient phylogenetic resolution was maintained to allow confirmation of the recombination event by examining the similarity existing between the minor parent and recombinant sequences represented by differences in the overall topology of the tree. It should be noted that although RDP3 can reliably predict the parental sequences that are involved in any recombination event, there is a noticeable lack of both sequence variation and phylogenetic separation in the lineage 1a sequences within the recombinant region. It is therefore possible that the minor parent may have been another lineage 1a sequence or a related ancestor; however, we are confident that the recombinant sequence (or its ancestor) was correctly identified.

## Discussion

The purpose of the present study was to examine a dataset consisting of multiple whole genome WNV sequences in order to determine the extent to which recombination contributed to the overall sequence variation within the this viral species and compare the contribution of recombination in WNV to that in other members of the *Flaviviridae *family.

We confirm the fact that WNV isolates can be grouped into 6 distinct phylogenetic clades or lineages [1,18]. Whether this implies that only 6 such lineages exist can only be confirmed with the acquisition of more sequence data. While the genetic differences producing these separate clades have apparently been produced as a result of geographic isolation, it is possible that temporal, host genetic, immune, and/or additional factors may also play some role in the generation of WNV diversity in these, or other replicating lineages.

Previous studies attempting to detect recombination in West Nile virus used only the envelope coding region [10]. For our current study, we hoped to increase the sensitivity of the analysis by utilizing the entire genome sequence for recombination detection. In spite of this, we were only able to detect one recombination event among all of the 154 WNV isolates that are available as complete genomic sequences. The NS5 region containing this recombination event is known to contain the WNV-specific loop/alpha-helix as well as the back subdomain of the RNA template tunnel [19].

Although recombination within certain species of the *Flavivirus *genus has been reported as fairly frequent--an observation which may likely be attributed to the vector-vertebrate host life cycle that is exploited by these arboviruses [10], it is not common across all species within the genus. Recombination is rare in Japanese encephalitis virus and St. Louis encephalitis virus, while recombination appears to be relatively frequent among the four serotypes of dengue virus with at least one known intergenotypic recombination event in serotype 1 [5,6,10]. Recombination also seems to be a relevant cause of genetic diversity within the *Hepatitis C virus *species (*Hepacivirus *genus). Such events have mostly been reported between genomes belonging to different genotypes or subtypes [7,20]; however, very few intra-subtype recombination events have been reported perhaps due to the difficulty of detecting recombination between very closely related viral genomes [21]. Since WNV is more closely related to Japanese encephalitis virus and St. Louis encephalitis virus than to either hepatitis C virus or dengue virus [22], its ability to utilize recombination as a mechanism for generating sequence variation may also be more limited.

We believe that this recombination event was identified because of the sequence variation existing between the two original parental lineages, and subsequently passed down through the progeny of the recombinant virus. Whether intra-lineage recombination is detectable is still unknown due to the high sequence similarity existing between such sequences. This idea is further supported by the previous observations that purifying selection pressure is present in arthropod-borne viruses [23], and that the sequence diversity present within the distinct lineages, and by extension, throughout the WNV species as a whole is remarkably low [24]. These arguments support our finding that the occurrence, and consequently the detection, of recombination within WNV is an especially rare event.

It is also important to realize that even though recombination was detected to have occurred between the SPU116_89_B and KN3829 sequences to yield the AnMg798 sequence, these are not likely the actual sequences that participated in the original recombination event. This statement is based on the knowledge that these sequences differ both in time and place of isolation, it is therefore probable that they are progeny of the original parental (and daughter) sequences. These extant sequences were likely flagged as having undergone a statistically significant recombination event due to the conservation of the original ancestral recombinant signal in the descendents.

Unfortunately, the sequence and metadata associated with these isolates is insufficient to determine the temporal or geographical point of origin for either the ancestral parental or daughter sequences. Therefore, while we know that the strains were isolated from eastern Africa, it is impossible to determine whether the ancestral parental strains were originally located adjacent to each other geographically or whether a bird, mosquito, human or other host infected with one of the parental strains migrated to an area where the second parental strain was either present or endemic. Either of these possibilities would result in the introduction of one of the parental strains into the same territory as the other and would allow for co-circulation of both viruses within the local environment until they eventually infected the same host and the recombination event occurred. It is also impossible with the present amount of information to determine which organism was co-infected and produced the recombinant virus.

There are several possible biological reasons why recombination may be so rare in WNV and therefore why we were only able to detect recombination in only 1 of the 154 WNV whole genome sequences. First, it has been shown that the concentration of WNV in the blood throughout the human portion of the replication cycle is low [25], which markedly decreases the probability that a single cell would become infected with the two distinct viral isolates required for recombination to occur. This is in contrast to infection in birds, the natural reservoir of WNV, which in some avian species can result in high levels of viremia [26]. So the possibility exists for a single avian cell to become infected by multiple strains of virus. Therefore the possibility remains for recombination to occur in birds (though if present, our analysis would have detected recombination within the available sequenced isolates irrespective of where recombination may have occurred). Secondly, it has also been shown *in vitro *that the WNV RNA polymerase is more likely to abort RNA replication after falling off of a template molecule than it is to reinitiate on a homologous RNA template [27]. This will decrease the likelihood of recombination in either the human or avian host.

## Conclusion

Using bioinformatics analysis, we were able to detect only a single incidence of recombination in available sequenced isolates of WNV. And in addition, reports indicate that the capability of the RdRp to template switch-and by extension to cause recombination-in WNV is severely diminished. For these reasons recombination appears not to be a likely mechanism for the generation of sequence diversity in West Nile virus.

## Methods

### Multiple Sequence Alignments and Phylogenetic Trees

To look for recombination in WNV isolates, we used 154 whole genome Kunjin virus and West Nile virus sequences (See additional file 3 for the original data used) obtained from the Viral Bioinformatics Resource Center . A multiple sequence alignment (MSA) of these genomes was constructed using MUSCLE [28]. Phylogenetic reconstruction of all available genomic sequences was performed using Bayesian analysis as implemented by the program MrBayes [29]. We used the default parameters in MrBayes (General Time Reversible evolutionary model, gamma-distributed rate variation and proportion of invariable sites) and sampled every 100 generations for 1 million generations using 4 chains. The first 2,500 trees were discarded as "burn-in".

### Recombination Analysis

For detection of recombination events, we used the automated suite of algorithms contained within the Recombination Detection Program 3 (RDP3) [8,30-38] to analyze the complete genomic sequences present in our MSA. In general, we used the default settings for each program in the RDP3 suite except for the following: for RDP we used a window size of 30; Bootscan used a window size of 200, step size of 50, and 50 bootstrap replicates; Siscan used a window size of 200 and step size of 20; and RDP3 was set to report all hits detected by 2 or more algorithms. In order to confirm the results from the automated tests, additional algorithms which are not part of the automated process were also run. SplitsTree4 [39] was used with default settings to assess the presence of a reticulated phylogenetic network as a representation of recombination (unpublished data).

## Competing interests

The authors declare that they have no competing interests.

## Authors' contributions

BP assisted in the design of the study, created the multiple sequence alignment, reconstructed the phylogenetic trees, performed the recombination analysis, and drafted the manuscript. EL conceived of and participated in the design and coordination of the study, and helped to draft the manuscript. All authors read and approved the final manuscript.

## Supplementary Material

Additional file 1 **RDP3 Screenshot of Positive Recombination Results**. Shows representative positive pairwise results from the RDP (top panel) and Bootscan (bottom panel) algorithms. Pairwise comparisons between the major and minor parents are shown in orange, between the minor parent and daughter sequence in purple, and between the major parent and the daughter sequence in blue. The area outlined in pink demarcates the region containing the recombinant signal. Click here for file

Additional file 2 **RDP3 Screenshot of Negative Recombination Results**. Shows representative negative pairwise results from the RDP (top panel) and SiScan (bottom panel) algorithms. Pairwise comparisons between the major and minor parents are shown in orange, between the minor parent and daughter sequence in purple, between the major parent and the daughter sequence in blue, and for the nearest outlier sequence in white. Click here for file

Additional file 3 **Table of Sequence Names and GenBank Accession Numbers Used in this Study**. additional table. Click here for file
