# Supplementary figures and images for: Recombination in West Nile Virus: minimal contribution to genomic diversity
Source: Virol J. 2009 Oct 12;6:165. doi: 10.1186/1743-422X-6-165 (PMC2763871; doi:10.1186/1743-422X-6-165)

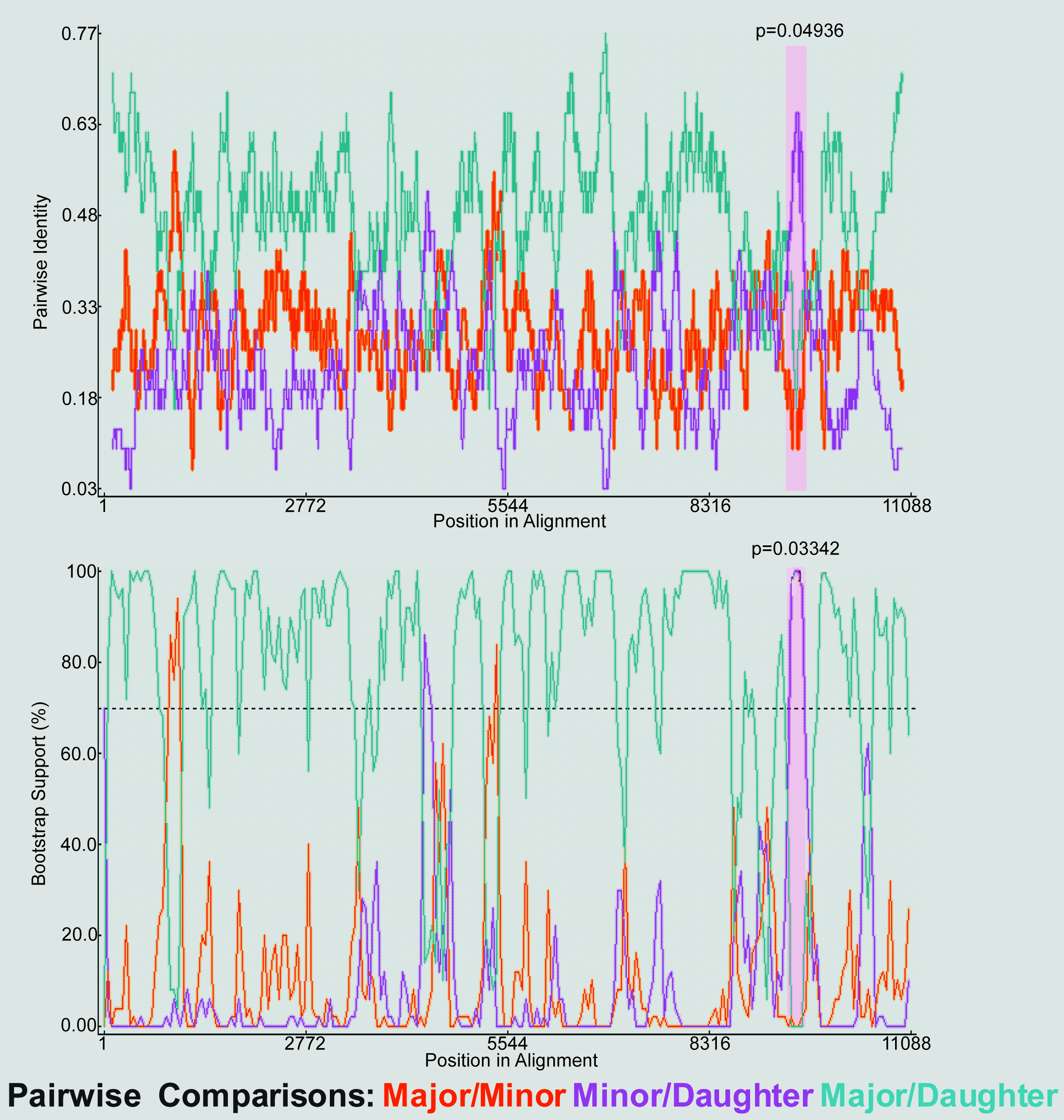

Supplement: Additional file 1 — RDP3 Screenshot of Positive Recombination Results. Shows representative positive pairwise results from the RDP (top panel) and Bootscan (bottom panel) algorithms. Pairwise comparisons between the major and minor parents are shown in orange, between the minor parent and daughter sequence in purple, and between the major parent and the daughter sequence in blue. The area outlined in pink demarcates the region containing the recombinant signal. [file 1743-422X-6-165-S1.TIFF]

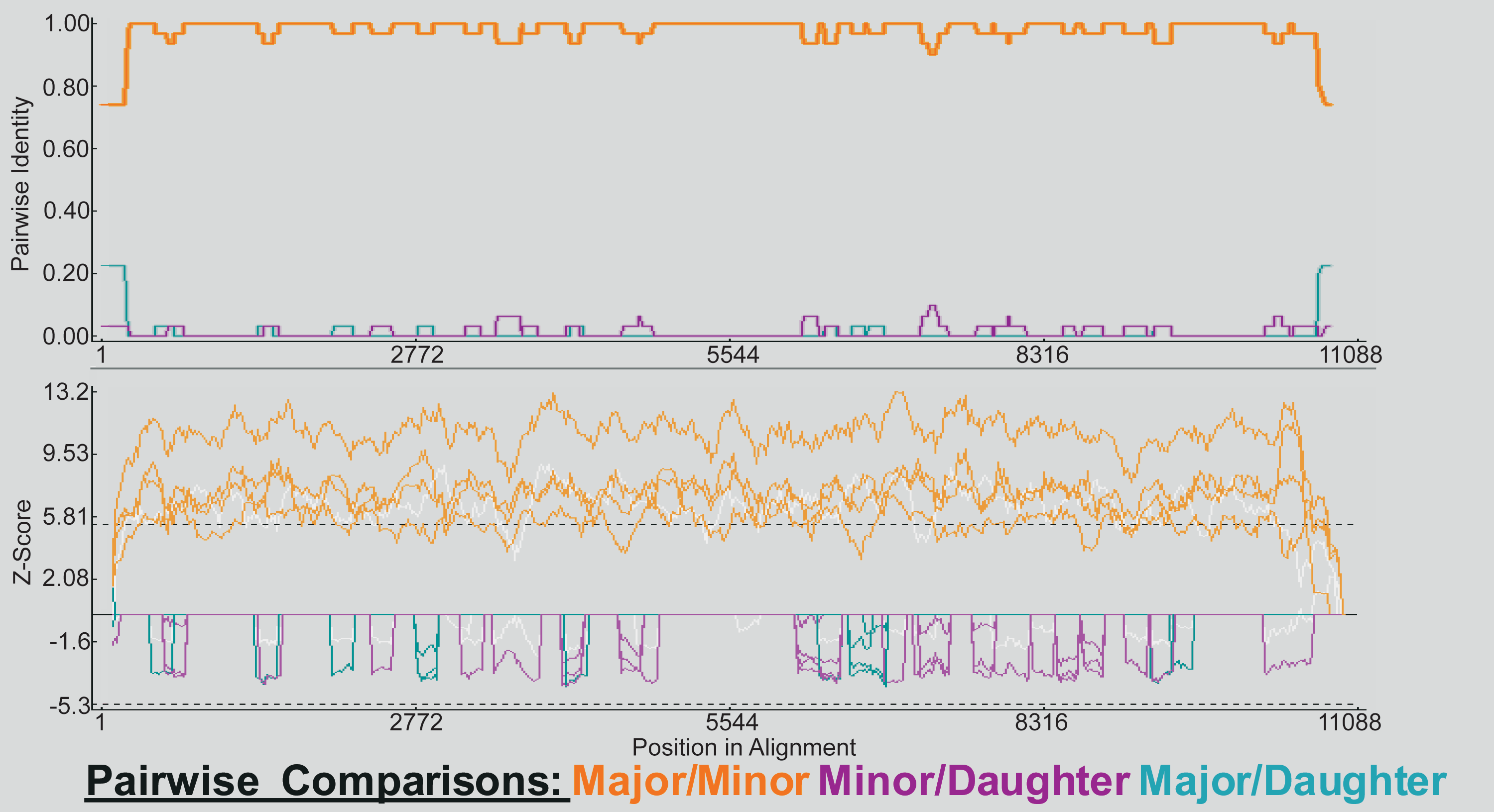

Supplement: Additional file 2 — RDP3 Screenshot of Negative Recombination Results. Shows representative negative pairwise results from the RDP (top panel) and SiScan (bottom panel) algorithms. Pairwise comparisons between the major and minor parents are shown in orange, between the minor parent and daughter sequence in purple, between the major parent and the daughter sequence in blue, and for the nearest outlier sequence in white. [file 1743-422X-6-165-S2.TIFF]
